# Existence of La-site antisite defects in $\hbox{LaMO}_3$ ($\hbox{M} = \hbox{Mn}$, Fe, and Co) predicted with many-body diffusion quantum Monte Carlo

**DOI:** 10.1038/s41598-023-33578-1

**Published:** 2023-04-25

**Authors:** Tom Ichibha, Kayahan Saritas, Jaron T. Krogel, Ye Luo, Paul R. C. Kent, Fernando A. Reboredo

**Affiliations:** 1 Materials Science and Technology Division, Oak Ridge National Laboratory, Oak Ridge, TN 37831 USA; 2 School of Information Science, Japan Advanced Institute of Science and Technology, Asahidai 1-1, Nomi, Ishikawa 923-1292 Japan; 3 Computational Sciences Division, Argonne National Laboratory, Argonne, IL 60439 USA; 4 Computational Sciences and Engineering Division, Oak Ridge National Laboratory, Oak Ridge, TN 37831 USA

**Keywords:** Theory and computation, Electronic properties and materials, Spintronics

## Abstract

The properties of $\hbox{LaMO}_3$ (M: 3*d* transition metal) perovskite crystals are significantly dependent on point defects, whether introduced accidentally or intentionally. The most studied defects in La-based perovskites are the oxygen vacancies and doping impurities on the La and M sites. Here, we identify that intrinsic antisite defects, the replacement of La by the transition metal, M, can be formed under M-rich and O-poor growth conditions, based on results of an accurate many-body ab initio approach. Our fixed-node diffusion Monte Carlo (FNDMC) calculations of $\hbox{LaMO}_3$ ($\hbox{M} = \hbox{Mn}$, Fe, and Co) find that such antisite defects can have low formation energies and are magnetized. Complementary density functional theory (DFT)-based calculations show that Mn antisite defects in $\hbox{LaMnO}_3$ may cause the *p*-type electronic conductivity. These features could affect spintronics, redox catalysis, and other broad applications. Our bulk validation studies establish that FNDMC reproduces the antiferromagnetic state of $\hbox{LaMnO}_3$, whereas DFT with PBE (Perdew–Burke–Ernzerhof), SCAN (strongly constrained and appropriately normed), and the LDA+*U* (local density approximation with Coulomb *U*) functionals all favor ferromagnetic states, at variance with experiment.

This manuscript has been authored by UT-Battelle, LLC, under contract DE-AC05-00OR22725 with the US Department of Energy (DOE). The US government retains and the publisher, by accepting the article for publication, acknowledges that the US government retains a nonexclusive, paid-up, irrevocable, worldwide license to publish or reproduce the published form of this manuscript, or allow others to do so, for US government purposes. DOE will provide public access to these results of federally sponsored research in accordance with the DOE Public Access Plan (http://energy.gov/downloads/doe-public-access-plan).

## Introduction

The $\hbox{LaMO}_3$ (M: 3*d* transition metal) perovskites display remarkable features such as superconductivity^1^, spin crossovers^2,3^, magnetic transitions, and giant magneto resistance^4–6^, and the transition metal atoms also provide redox catalytic ability^7^. Beyond the basic science interests in perovskites, their properties are exploited for various applications, including gas sensors^8,9^, thermal sensors^2^, sub-micrometer magnetic field sensors^10^, photocatalysts, catalytic combustion^11^, air batteries^12^, magnetic read heads, and magnetic memory cells^13^.

More interestingly, perovskites can be grown on top of each other via pulsed laser deposition (PLD) and molecular beam epitaxy (MBE)^14,15^. These growth methods allow the fabrication of artificial materials that combine the properties of the individual building blocks with additional effects that result from the interfaces and strain caused by lattice mismatch. For example, strain affects the magnetism^16^, electronic conductivity^17^, ferroelectricity^18^, carrier density^17^, and oxygen vacancy concentration^19^. Interestingly, 2D superconductivity is observed at the two types of interfaces of $\hbox{LaAlO}_3/\hbox{SrTiO}_3$ and $\hbox{LaTiO}_3/\hbox{SrTiO}_3$^20,21^. The relative lower temperatures in PLD and MBE facilitates the formation of defects as a result of departures of the stoichiometry or limited annealing times. Because defects are well known to alter the magnetic, electronic, and chemical properties of perovskites, their characterization is key to understanding this family of materials and the composite materials derived from them. At equilibrium, the formation energy of defects determines their relative abundance. However, outside this regime, the relative abundance of defective structures also depends on growth kinetics. Defects with high formation energies will be difficult to form even out of equilibrium. Therefore, the formation energy of defects is a key indicator of their occurrence during natural or artificial growth.

For general $\hbox{ABO}_3$ perovskites, defects are theoretically possible on any of the atomic sites and in defect complexes. In La-based perovskites, a strong focus has been on the formation energy of the oxygen vacancies because these play an important role in the oxygen reduction reaction^22^, oxygen evolution reaction^23^, and ionic conduction^24,25^. However, in non-La-based perovskites, such as $\hbox{YAlO}_3$ and $\hbox{LuAlO}_3$, both Y and Lu antisite defects have been predicted^26^. Similarly, DFT calculations predicted that Ni impurities in $\hbox{BaZrO}_3$ occupy the A-site under Ni-rich condition^27^. Experiments also found that Y impurities, B-site dopants in $\hbox{BaZrO}_3$, occupy the A-site as well in the Y richer phase^28^. These examples raise the question as to the extent La-site antisite defects are relevant in $\hbox{LaMO}_3$ perovskites.

Transition metal oxides, including perovskites, are notoriously challenging to current density functional theory (DFT) approximations^29^ because strong static and dynamic electronic correlations and self-interaction errors are present at the partially occupied *d* shells^30^. These errors are compounded for defects that involve transition metals. Accordingly, these materials and their defects are important targets of quantum many-body methods, such as fixed-node diffusion Monte Carlo (FNDMC), that account for electronic correlations and avoid the self-interaction error^31,32^. These methods can be applied to defective supercells and to the ideal bulk solids, making them well-suited to modeling transition metal oxides in general^33–50^.

In this paper, we study the formation energy of antisite defects and oxygen vacancies in $\hbox{LaMO}_3$ (M = Mn, Fe, and Co) using FNDMC. We establish for $\hbox{LaMnO}_3$ and $\hbox{LaFeO}_3$ that the formation energy of antisite defects is low enough to form the defects under M-rich and O-poor chemical conditions. However, in $\hbox{LaCoO}_3$, the formation energy of antisite defects is higher for each type of growth conditions studied here. We also show that the antisite defects, as well as the oxygen vacancies, significantly affect the properties of La perovskites. The predicted partial density of states (PDOS) suggests that the antisite defect formation may contribute to the *p*-type electronic conductivity in $\hbox{LaMnO}_3$ and may narrow the band gap of $\hbox{LaFeO}_3$ and $\hbox{LaCoO}_3$. In addition, we study the magnetic energy order of the non-defective perovskite crystals. Because even the magnetic ground state is still controversial for some perovskites, we determine the magnetic ground state prior to conducting the defect studies. For $\hbox{LaMnO}_3$ and $\hbox{LaFeO}_3$, FNDMC corroborates the experimental antiferromagnetic (AFM) ground state, but the ground state of pristine $\hbox{LaCoO}_3$ remains controversial^16^.

The rest of the paper is organized as follows: In “Calculation details” section, we explain how the point defect formation energies are evaluated, including the details of the DFT and FNDMC calculations. In “Results and discussion” section, we discuss the magnetic state of non-defective perovskite crystals. We also discuss the defect formation energies and how the point defects affect electronic conductivity. This work is summarized in “Conclusion” section.

## Calculation details

### Formation energy of defects

The formation energies of oxygen vacancy $\hbox{V}_{\textrm{O}}$ and intrinsic transition metal antisite defects on the La site $\hbox{M}_{\textrm{La}}$ (M = Mn, Fe, and Co) were evaluated in the neutral state by using the following equations:

1 $$\begin{aligned} \Delta E\left( {\mathrm{{V}}_{\mathrm{{O}}} } \right)= & {} E_{\textrm{V}_{\textrm{O}} } - {E_{\mathrm{{bulk}}}} + {\mu _{\mathrm{{O}}}}, \end{aligned}$$

2 $$\begin{aligned}\Delta E\left( {{\text{M}}_{{\text{La}}}}\right)=E_{{\text{M}}_{\text{La}}} - E_{\text{bulk}} + \mu_{{\text{La}}} - \mu _{\text{M}}. \end{aligned}$$

Here, ${E_{\mathrm{{bulk}}}}$ is the total energy of perovskite supercells with no defects, $E_{\textrm{V}_{\textrm{O}}}$ is an isolated oxygen vacancy, $E_{\textrm{M}_{\textrm{La}}}$ is an isolated antisite defect, and ${\mu _{\mathrm{{X}}}}$ is the chemical potential of the atomic species $\textrm{X}$. The formation of charge-neutral oxygen vacancy reduces the neighboring cations. The influence of charge-neutral antisite defect formation on the neighboring ions is discussed in “Atomic distortions around the antisite defect” section. The effects of electron and hole doping are discussed in the supplemental information (SI). The chemical potential (${\mu _{\mathrm{{X}}}}$) and the defect formation energies ($\Delta E\left( {\mathrm{{V}}_{\mathrm{{O}}} } \right)$ and $\Delta E\left( {\mathrm{{M}}_{{{\textrm{La}}}} } \right)$) for different equilibrium states are characterized by the solids or gases present during growth. The calculated total energies for the materials were used to determine the chemical potentials to simulate several equilibrium states and are listed in Table 1.

**Table 1:** List of compounds used to calculate the chemical potentials in each equilibrium state.

Formula	Space group	Lattice constants (Å)	Magnetic state	Supercell size	
$\hbox{O}_2$	–	$d=1.24$ (molecule)	–	–	
$\hbox{La}_2\,\hbox{O}_3^{1}$	*C*2/*m*	$a=b=3.93$, $c=6.15$^52^	NM	16	(80 atoms)
$\hbox{LaMnO}_3$	*Pnma*	$a=5.54$, $b=5.75$, $c=7.69$^53^	AFM-A^54^	4	(80 atoms)
MnO	$Fm{\bar{3}}m$	$a=b=c=4.49$^55^	AFM-A$^{2}$	40	(80 atoms)
$\hbox{MnO}_2$	$P4_2/mnm$	$a=b=4.36$, $c=2.88$^54^	AFM-A^54^	13	(78 atoms)
$\hbox{LaFeO}_3$	*Pnma*	$a=b=5.56$, $c=7.85$^56^	AFM-G^57^	4	(80 atoms)
Fe	$Im{\bar{3}}m$	$a=b=c=2.87$^58^	FM	64	(64 atoms)
FeO	$Fm{\bar{3}}m$	$a=b=c= 4.334$^59^	AFM-A$^{2}$	36	(72 atoms)
$\hbox{Fe}_2\,\hbox{O}_3$	$R{\bar{3}}c$	$a=b=5.04$, $c=13.75$^60^	AFM^61^	8	(80 atoms)
$\hbox{La}_2\,\hbox{O}_3^{3}$	$Ia{\bar{3}}$	$a=b=c=11.40$^62^	NM	Extrap.$^{4}$	
Co	$P6_3/mmc$	$a=b=2.47$, $c=4.02$^63^	FM	Extrap.$^{4}$	
CoO	$F {\bar{4}} 3 m$	$a=b=3.20$, $c=7.70$^64^	AFM^64^	Extrap.$^{4}$	
$\hbox{Co}_3\,\hbox{O}_4$	$Fd{\bar{3}}m$	$a=b=c=8.05$^65^	FM	Extrap.$^{4}$	
$\hbox{LaCoO}_3$	$R {\bar{3}} c$	$$\begin{array}{l} a= 5.36, b= 5.44, c= 7.62, \\ \alpha =89.97^\circ , \beta =88.83^\circ , \gamma =89.79^\circ \end{array}$$^66^	AFM-G^66^	Extrap.$^{4}$	

This table summarizes the lattice constants, the magnetic state, and the supercell size as number of primitive cells and atoms. ($^{\textrm{a}}$ For $\hbox{LaMnO}_3$ and $\hbox{LaFeO}_3$ results; $^{\textrm{b}}$ The energy differences between different AFM structures of the rock-salt type MnO were calculated to be within 50 meV per formula unit by DFT^51^. This energy scale is significantly small compared with the energy scale of point defects formation: the choice of the spin structure would not be significant for the point defects formation energies; $^{\textrm{c}}$ For $\hbox{LaCoO}_3$ results; $^{\textrm{d}}$ The total energy was evaluated by the size extrapolation. See the SI for the details).

### Relaxation of defective and bulk structures

The Vienna Ab Initio Simulation Package (VASP)^67^ was used to relax the atomic positions. The total energy and orbital eigenenergies convergence criteria for the self-consistent field (SCF) process were both $1\times 10^{-5}$ eV/simulation cell. The atomic positions were relaxed until the maximum residual force was less than 0.01 eV/Å. We found that the defect formation energy does not significantly change when the structure is altered to the one obtained with a different functional choice (details available in the SI). The lattice vectors were fixed at the reference values listed in Table 1. For $\hbox{LaCoO}_3$, we used the same calculation settings as our previous work^16^. To calculate the chemical potentials and cohesive or formation energies of bulk structures, the atomic positions were also fixed at the reference data values in Table 1. For $\hbox{LaMnO}_3$ and $\hbox{LaFeO}_3$, we used the Perdew–Burke–Ernzerhof (PBE) functional^68^ to relax the atomic positions of both bulk and defective structures. The core electrons were replaced using the projector augmented wave (PAW) method^69^. The plane-wave cutoff energy was 520 eV, which converged the total energy of $\hbox{LaMnO}_3$ within 14 meV/atom. The *k*-mesh spacing was smaller than 0.50 Å$^{-1}$, which converged the total energy of $\hbox{LaMnO}_3$ within 2.4 meV/atom. The same calculation settings were used to obtain the $\hbox{LaMnO}_3$, $\hbox{LaFeO}_3$, and $\hbox{LaCoO}_3$ AFM and FM energy differences. The cohesive or formation energies of the bulk systems listed in Table 1 were calculated using PBE^68^ and strongly constrained and appropriately normed (SCAN) functionals^70^.

### FNDMC calculations’ details

We performed FNDMC calculations with the high-performance QMCPACK code^71,72^ with the Nexus workflow management software^73^. We used the Slator-Jastrow-type trial wave functions^74^. The Jastrow factor consisted of one-, two-, and three-body terms. The orbitals of the Slater determinants were obtained with the local density approximation with Coulomb interaction potential (LDA+*U*) method^30^. Further details of the LDA+*U* calculations are written in the next subsection. The time step was $dt=0.01$ a.u.$^{-1}$, and the associated errors were 5 meV/atom for $\hbox{LaMnO}_3$ and $\hbox{LaFeO}_3$^54,75^ and less than 20 meV/atom for $\hbox{LaCoO}_3$^16^. The target population of walkers was 2000 or larger for our main results (the SI discusses a few exceptions). We used twist-averaged boundary conditions and size extrapolation to estimate the one- and two-body finite size effects (details in the SI). For $\hbox{LaFeO}_3$, $E_{{\textrm{V}}_{\textrm{O}}}$ and $E_{\textrm{bulk}}$ in Eq. (1) were taken from our previous FNDMC results^75^.

### Tuned LDA+*U* trial wave function

We used the Quantum ESPRESSO package^76^ to run the LDA+*U* calculations. We used the norm conserving pseudopotentials^54,75,77^, whose accuracy has been verified in our previous works^54,75,77^. The cutoff energy was 350 Ry, which converged the total energy of $\hbox{LaCoO}_3$ within 1 meV/atom. The *k*-mesh size was identical to the twist-averaging mesh size (details in the SI). The energy convergence criterion for the SCF process was 5$\times 10^{-6}$ Ry or smaller. The Hubbard *U* contribution was applied to the 3*d* electrons of Mn, Fe, and Co. We optimized the *U* value for Mn (Fe) to minimize the FNDMC total energy of the bulk $\hbox{LaMnO}_3$ ($\hbox{LaFeO}_3$): $U_{\textrm{opt}}=3$ eV for Mn and 6 eV for Fe. We optimized the *U* value for Co to minimize the FNDMC total energy of every bulk system: $U_{\textrm{opt}}=6$ eV for $\hbox{LaCoO}_3$ and Co and $U_{\textrm{opt}}=5$ eV for CoO and $\hbox{Co}_3\,\hbox{O}_4$. We consistently used the $U_{\textrm{opt}}$ values for our LDA+*U* calculations throughout the paper. We also used LDA+*U* with the optimal *U* values to obtain the PDOS of the perovskites because the FNDMC tuning of DFT+*U* has been reported to improve the reliability of DFT to study physical properties^40,78,79^.

## Results and discussion

### Cohesive or formation energies and energy differences of magnetic states given by FNDMC and DFT

**Figure 1:**
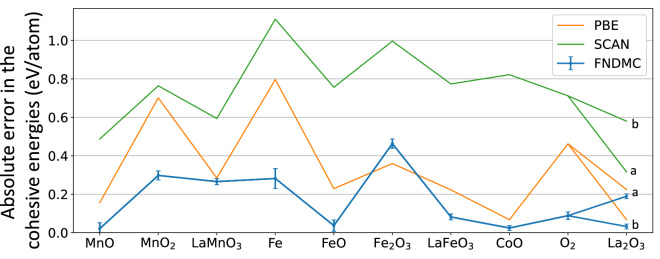
Cohesive energies of the different compounds used to evaluate the chemical potentials in this study compared with experimental data (zero value). a: For $\hbox{LaMnO}_3$ and $\hbox{LaFeO}_3$ results. b: For $\hbox{LaCoO}_3$ results.

The total energies of the materials listed in Table 1 were calculated to obtain the chemical potentials, $\mu _{\textrm{La}}$, $\mu _{\textrm{M}}$, and $\mu _{\textrm{O}}$, for different chemical equilibrium conditions. The chemical potentials were used to calculate the defect formation energies with Eqs. (1) and (2). To verify the results, the calculated cohesive energies were compared with the available experimental data^24,80,82–89^ (Table S1 in the SI). The differences between calculated and experimental cohesive energies are shown in Fig. 1. The experimental and FNDMC numerical values are listed in Table 1 in the SI. To quantitatively assess the reliabilities of different methods, the mean squared deviations (MSD) were calculated from the experimental data for the cohesive energies. The MSDs were 0.046(4) (eV/atom)$^2$ for FNDMC, 0.158 (eV/atom)$^2$ for PBE, and 0.562 (eV/atom)$^2$ for SCAN: FNDMC gave the lowest MSD. Because the experimental cohesive energies were not found for $\hbox{Co}_3\hbox{O}_4$ and $\hbox{LaCoO}_3$, we alternatively compared the formation energies in Table 2: FNDMC reproduced the experimental values significantly better than the DFT approximations that were considered.

**Table 2:** Experimental and calculated formation enthalpies (eV/atom) of $\hbox{Co}_3\hbox{O}_4$ and $\hbox{LaCoO}_3$. The last row indicates the mean of the squared deviations (MSD) from the experimental values.

	Expt.	FNDMC	PBE	SCAN
$\hbox{Co}_3\,\hbox{O}_4$	1.33^80^	1.28 (1)	1.00	1.06
$\hbox{LaCoO}_3$	2.55^81^	2.63 (1)	2.23	2.64
MSD	–	0.004 (1)	0.112	0.045

We also calculated the energy differences between FM and AFM states of the perovskites. These differences are listed in Table 3. A negative (positive) value indicates that the AFM (FM) state is more stable. For both $\hbox{LaMnO}_3$ and $\hbox{LaFeO}_3$, the AFM ground state was reported experimentally^53,90^. Our FNDMC calculations reproduced the AFM ground state for both materials. For $\hbox{LaFeO}_3$, the functionals all reproduced the AFM ground state. SCAN agrees well with FNDMC (FMDMC: −0.08(1) vs. SCAN: −0.08 eV). However, none of the DFT functionals that we tested gave the AFM ground state for $\hbox{LaMnO}_3$.

**Table 3:** Total energy difference (eV/f.u.) between AFM and FM states of $\hbox{LaMnO}_3$, $\hbox{LaFeO}_3$, and $\hbox{LaCoO}_3$: the total energy of the AFM state minus that of the FM state. Negative values indicate greater AFM stability.

Perovskite	PBE	SCAN	LDA+*U*	FNDMC
$\hbox{LaMnO}_3$	+ 0.02	+ 0.07	+ 0.10	− 0.15 (2)
$\hbox{LaFeO}_3$	− 0.16	− 0.08	− 0.23	− 0.08 (1)
$\hbox{LaCoO}_3$	+ 0.50	− 0.18	− 0.37	− 0.27 (2)^16^

Determining the magnetic ground state for $\hbox{LaCoO}_3$ is rather more complex than for $\hbox{LaMnO}_3$ and $\hbox{LaFeO}_3$ because different spin states of the cobalt ion are nearly degenerated. Here, we briefly discuss the main results of our previous work^16^. The ground state of bulk $\hbox{LaCoO}_3$, $\hbox{Co}^{3+}$, was reported experimentally in 1957 to be low spin (LS) $t^6_{2g}e^0_{g}$ at low temperature (T<30 K) and therefore non-magnetic (NM)^91^. However, experiments in recent years have challenged this idea^92–96^. It is argued that at elevated temperatures, the LS $\hbox{Co}^{3+}$ transitions into a high-spin/low-spin mixture; at temperatures above 500K, the ground state is completely high-spin (HS; $t^4_{2g}e^2_{g}$) $\hbox{Co}^{3+}$^91^. In our FNDMC calculations, we found that the ground state of $\hbox{LaCoO}_3$ at 0 K is an HS AFM^16^ state. Using FNDMC, the magnetic state energy ordering was revealed to be HS-AFM < HS/LS-FM < HS-FM < LS < intermediate spin-FM. The FNDMC total energy difference between the most and second-most stable states (i.e., HS-AFM and HS/LS-FM) was 0.15 eV, which indicates an HS-AFM ground state. Table 3 shows the energy differences of HS-AFM−HS/LS-FM. SCAN and LDA+*U* reproduce FNDMC; PBE does not.

From the above discussion, we conclude that FNDMC is better at evaluating the energies related to the perovskite systems. Therefore, we used FNDMC to evaluate the defect formation energies.

### Local magnetization of point defects

**Figure 2:**
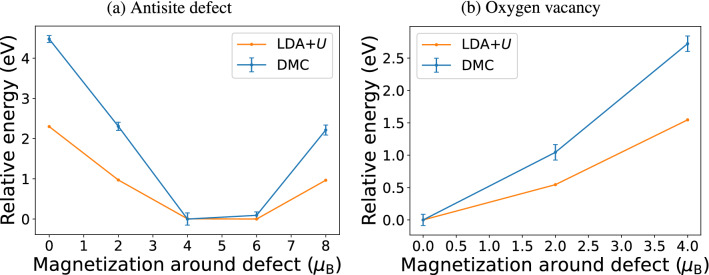
(Color Online) Relative energies obtained with FNDMC (blue) and LDA+*U* (orange) calculations of $\hbox{LaMnO}_3$ with (**a**) antisite defect and (**b**) oxygen vacancy as a function of the total magnetization. The lowest data point is set to zero. LDA+*U* reproduces the defect magnetization (i.e., energy minima) of FNDMC.

A point defect was introduced into the bulk supercell with the AFM ordering because this is the ground state of the bulk structures and we target the formation energy of an isolated point defect. We optimized the magnetic moment around the point defect to minimize the total energy. Figure 2 shows the total energies of defects in $\hbox{LaMnO}_3$, antisite defects or oxygen vacancies, for different magnetic moments around the defect. The blue lines are the FNDMC results, and the orange lines are the LDA+*U* results. The total energies are shown as the relative differences from the lowest value. The minima of FNDMC and LDA+*U* agreed with each other. The antisite defect was magnetized by 4 or 6 $\mu _{\textrm{B}}$ in $\hbox{LaMnO}_3$, whereas the oxygen vacancy is not magnetized.

Because FNDMC and LDA+*U* agreed with each other in terms of the magnetization of point defects in $\hbox{LaMnO}_3$, we simply used LDA+*U* to determine the magnetization of defects used for the FNDMC calculations for $\hbox{LaFeO}_3$ and $\hbox{LaCoO}_3$. For $\hbox{LaFeO}_3$, we obtained 5 $\mu _{\textrm{B}}$/defect for the antisite defect and 0 $\mu _{\textrm{B}}$/defect for the oxygen vacancy. For $\hbox{LaCoO}_3$, we obtained 4 $\mu _{\textrm{B}}$/defect for the antisite defect and 0 $\mu _{\textrm{B}}$/defect for the oxygen vacancy. In all the perovskites, the transition metal antisite defects have finite local magnetizations, but the oxygen vacancy does not.

### Relative abundance of antisite defects in $\hbox{LaMnO}_3$ and $\hbox{LaFeO}_3$

**Figure 3:**
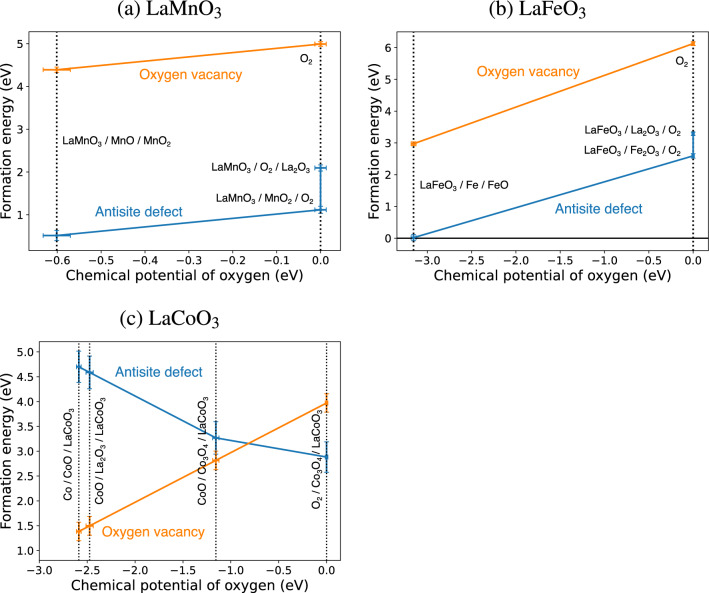
FNDMC prediction of the antisite defect and the oxygen vacancy formation energies as a function of O chemical potential for (**a**) $\hbox{LaMnO}_3$ (**b**) $\hbox{LaFeO}_3$, and (**c**) $\hbox{LaCoO}_3$. The vertical lines indicate the chemical potential where three compounds coexist.

**Figure 4:**
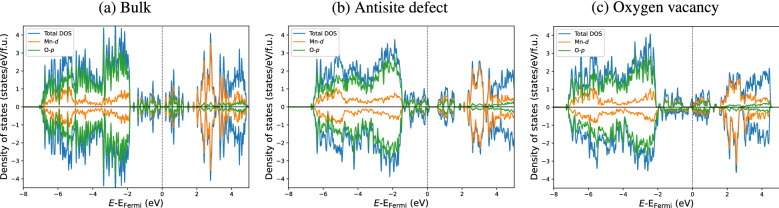
Density of states of $\hbox{LaMnO}_3$ with (**a**) no defects (bulk), (**b**) antisite defects, and (**c**) oxygen vacancies.

Figure 3 illustrates the main result of this research: the antisite defect and oxygen vacancy formation energies of $\hbox{LaMO}_3$ ($\hbox{M} = \hbox{Mn}$, Fe, and Co) for different chemical equilibrium conditions. In the case of $\hbox{LaMnO}_3$ and $\hbox{LaFeO}_3$, the antisite defect formation energies are almost always significantly lower than the oxygen vacancy formation energies. For $\hbox{LaMnO}_3$, a very small antisite defect formation energy (0.51(12) eV) was predicted at the chemical potentials, where MnO, $\hbox{MnO}_2$, and $\hbox{LaMnO}_3$ coexist. Similarly, in the case of $\hbox{LaFeO}_3$, the antisite formation energy at the O-poor condition limit, where $\hbox{LaFeO}_3$,Fe, and FeO coexist, was predicted to be almost zero (0.016(95) eV). The antisite defect formation energies in $\hbox{LaCoO}_3$ are always high (> 2.5 eV), and the formation of antisite defects at equilibrium appears to be very difficult. In summary, our results suggest possible antisite defect formation in $\hbox{LaMnO}_3$ and $\hbox{LaFeO}_3$ for M-rich and O-poor conditions because the formation energy appears to be much lower than the oxygen vacancy (Fig. 4) that is often reported in perovskite materials.

Table 4 summarizes the formation energies of the oxygen vacancy at the O-rich limit (i.e., $\mu _{\textrm{O}} = 0.5 \cdot E(\textrm{O}_{2})$) obtained with different methods. Our FNDMC calculations nearly reproduced experimental estimates of the oxygen vacancy formation energies for $\hbox{LaMnO}_3$ and $\hbox{LaFeO}_3$. This corroborates the accuracy of our FNDMC calculations for defects. Among the DFT results, the PW91+*U* method also nearly reproduced the experimental results^97,98^, but the others did not. These previous DFT calculations without the Hubbard *U* correction overestimated the oxygen vacancy formation energies for the $\hbox{LaMnO}_3$ case. The Hubbard *U* correction tends to decrease the vacancy formation energy. For the $\hbox{LaCoO}_3$ case, the previous DFT calculations with the Hubbard *U* correction all underestimated the oxygen vacancy formation energy compared to our FNDMC results.

**Table 4:** Comparison of oxygen vacancy formation energies at the most O-rich condition.

Reference	$\hbox{LaMnO}_3$	$\hbox{LaFeO}_3$	$\hbox{LaCoO}_3$
FNDMC	4.20 (5) eV	6.12 (6) eV	3.97 (19) eV
Expt.	3.6–4.1 eV^97,99,100^	4–6 eV^97,101^	–
PW91+*U*	3.6 eV^97,98^	4.1–4.5 eV^97,98^	2.7 eV^97,98^
PBE+*U*	–	–	2.23 eV^102^
LDA+*U*	3.14 eV^103^	–	3.07 eV^102^
PW91	4.7 eV^104^	–	–
PBE	$\sim$4.5 eV^105^	–	–

Our FNDMC calculations suggested a relative abundance of antisite defects; however, no direct observations of antisite defects were found in the literature review. This lack could be due to the difficulty in observing these antisite defects. Because the transition metal atom has fewer electrons (25,26, and 27) than the La atom (57), the antisite defects would be masked by the La atom and cannot be easily observed in the transmission electron microscopy experiments. Similarly, x-ray diffraction experiments would not observe the antisite defects unless they are ordered. These reliable FNDMC results of antisite defects formation could accelerate their discovery in perovskites.

### Atomic distortions around the antisite defect

**Figure 5:**
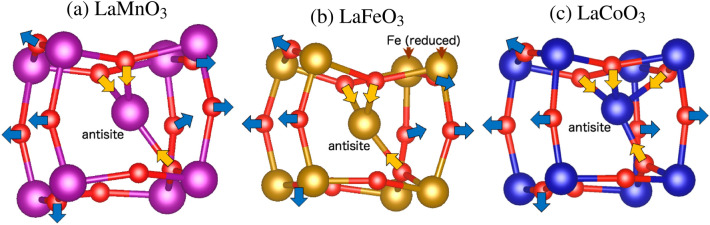
Atomic distortions around the antisite defect in $\hbox{LaMO}_3$ (M=Mn, Fe, and Co).

Figure 5 shows the relaxed structure around the antisite defect in $\hbox{LaMO}_3$ (M=Mn, Fe, and Co). The antisite defect shifts from the original La site position towards some of the surrounding oxygen atoms. This is attributed to the significantly smaller ionic radii of $\hbox{Mn}^{3+}$, $\hbox{Fe}^{3+}$, and $\hbox{Co}^{3+}$ (respectively 0.785, 0.785, and 0.75 Å) compared to that of $\hbox{La}^{3+}$ (1.172 Å)^106^. Table 5 compares the distances between the antisite defect and surrounding O atoms with those between the La atom and surrounding O atoms in the bulk structure. This table clarifies that the antisite defect selectively bonds with some specific O atoms compared to La atoms, because of the shorter ionic radius than La. The coordination numbers of O atoms around the antisite defect appears to be three for $\hbox{LaMnO}_3$ and $\hbox{LaFeO}_3$ and four for $\hbox{LaCoO}_3$ based on the listed distances.

**Table 5:** Bonding distances $d_{\textrm{bonding}}$ (Å) between La atom and surrounding O atoms in bulk $\hbox{LaMO}_3$ (left column) and antisite defect and surrounding O atoms in $\hbox{LaMO}_3$ (right column) for M $=$ Mn, Fe, and Co, respectively. Up to the 6th shortest bonding distances are listed in ascending order. The bottom row indicates the sum of ionic radii $d_{\textrm{ionic}}$ (Å) of the bonding ions: $\hbox{La}^{3+}$ or $\hbox{M}^{3+}$ and $\hbox{O}^{2-}$^106^.

	$\hbox{LaMnO}_3$		$\hbox{LaFeO}_3$		$\hbox{LaCoO}_3$	
	La–O	$\hbox{Mn}_{La}$–O	La–O	$\hbox{Fe}_{La}$–O	La–O	$\hbox{Co}_{La}$–O
$d_{\textrm{bonding}}$	2.427	2.051	2.395	1.915	2.409	1.849
	2.470	2.071	2.426	1.923	2.409	1.899
	2.470	2.089	2.426	1.923	2.409	1.935
	2.546	2.450	2.526	2.592	2.768	1.966
	2.625	2.928	2.647	2.715	2.768	2.725
	2.625	2.945	2.647	3.072	2.768	2.822
$d_{\textrm{ionic}}$	2.57	2.19	2.57	2.19	2.57	2.15

We next discuss the formal charges of the antisite defects comparing the antisite–O distances and the antisite’s coordination numbers with different transition metal (TM) oxides’ TM–O distances and TM’s coordination numbers. The TM–O distances and TM’s coordination numbers are summarized in Table 6. The TM–O distances are smaller or TM’s coordination number is larger when the TM’s formal charge is larger. For the $\hbox{LaMnO}_3$ case, the antisite–O distances are slightly larger than the TM–O distances in the bulk $\hbox{LaMnO}_3$, and the antisite’s coordination number is also smaller. Therefore, the antisite defect’s formal charge is smaller than $+$3. For the $\hbox{LaFeO}_3$ case, whereas the antisite–O distances are shorter than the TM–O distances of bulk $\hbox{LaFeO}_3$ and $\hbox{Fe}_2\,\hbox{O}_3$, the antisite’s coordination number is smaller. Therefore, the formal charge of the antisite defect cannot be decided based on distances and coordination. Similarly, the antisite’s formal charge in $\hbox{LaCoO}_3$ cannot be decided: the antisite–O distances are smaller than the TM–O distance in the bulk $\hbox{LaCoO}_3$ but the antisite’s coordination number is smaller.

**Table 6:** The formal charges $Q_{\textrm{formal}}$ and Bader charges $Q_{\textrm{Bader}}$ of transition metal (TM) ion, distances between the TM and nearest O ions, and O coordination numbers around a TM ion in different transition metal oxides. The parenthesis value indicates the number of bonds of the bonding distance.

System	$Q_{\textrm{formal}}$	$Q_{\textrm{Bader}}$	TM-O distances (Å)
MnO	+ 2	+ 1.36	$2.25(\times 6)$
$\hbox{LaMnO}_3$	+ 3	+ 1.67	$1.97(\times 2)$, $1.99(\times 2)$, $2.08(\times 2)$
$\hbox{MnO}_2$	+ 4	+ 1.89	$1.88(\times 6)$
FeO	+ 2	+ 1.34	$2.17(\times 6)$
$\hbox{Fe}_2\,\hbox{O}_3$	+ 3	+ 1.84	$1.93(\times 3)$, $2.14(\times 3)$
$\hbox{LaFeO}_3$	+ 3	+ 1.81	2.00–$2.02(\times 6)$
CoO	+ 2	+ 1.21	$+1.96 (\times 4)$
$\hbox{LaCoO}_3$	+ 3	+ 1.63	$2.00(\times 6)$

In order to estimate the formal charge of the antisite defects, we calculated their Bader charges^107–110^ for the electronic densities given by LDA+*U* method. For the $\hbox{LaMnO}_3$ case, Mn atoms in the bulk structure have larger Bader charge (1.67 e) than the antisite defect’s (1.43 e). This supports the discussion in the prior paragraph that the antisite defect’s formal charge is smaller than +3. For the $\hbox{LaFeO}_3$ case, the antisite defect has the same Bader charge as the Fe atoms’ in the bulk structure (1.78 e). However, the Fe atoms labeled “(reduced)” in Figure 5 have smaller Bader charges (1.59 e): their formal charges are smaller than +3. On the other hand, for the $\hbox{LaCoO}_3$ case, the antisite and the other Co ions have similar Bader charges to the Co atoms’ in the bulk $\hbox{LaCoO}_3$.

The Bader charges of La atoms in the bulk $\hbox{LaMO}_3$ ($\sim$2.14 e) are significantly larger than the antisite defects’. Therefore, the antisite defects could disturb the local charge neutrality. Positively charged antisite defects could be more easily formed when the Fermi energy is lower. It remains to be investigated in the future how the antisite defect formation energies depend on the defect charge and Fermi energy. The neutral defects do not depend on the Fermi energy and are thus an upper bound for the formation energies of these defects. While very high or very low Fermi energies may lower the energies of charge defects below the neutral ones, the existence of charge defects will not change the main conclusion of this work since it can lower their formation energy further.

### Defect’s contributions to the density of states

Figure 4 shows the PDOS of $\hbox{LaMnO}_3$ without defects, with antisite defects, and with oxygen vacancies. The PDOS of $\hbox{LaFeO}_3$ and $\hbox{LaCoO}_3$ are given in the SI. The total magnetizations were obtained in DFT, without the restriction used in FNDMC that constrains the magnetization value to be an integer. We obtained different total magnetization from the trial wave functions for $\hbox{LaMnO}_3$ with antisite defects (4$\rightarrow$5.05 $\mu _{\textrm{B}}$) and for $\hbox{LaMnO}_3$ with oxygen vacancies (0$\rightarrow$0.15 $\mu _{\textrm{B}}$). However, the energy differences were less than 0.016 eV/atom.

LDA+*U* with the optimal *U* values yielded by FNDMC reproduced earlier reports that the bulk $\hbox{LaMO}_3$ (M = Mn, Fe, and Co) are insulators. However, the $\hbox{LaMnO}_3$ band gap given by LDA+*U* was 0.08 eV, which is significantly smaller than the experimental values, 1.7 and 1.9 eV^111,112^. In our previous work, PBE+*U* also underestimated the band gap (0.2 eV) and FNDMC reasonably reproduced the experimental value (2.3(3) eV)^54^. DFT with a hybrid functional also gave 2.3 eV^113^ so the Hubbard *U* correction alone would not be enough to obtain the band gaps of $\hbox{LaMnO}_3$. The $\hbox{LaFeO}_3$ band gap given by LDA+*U* was 2.77 eV, which is close to a reported value of 2.37 eV, which was produced by applying Tauc models to experimental data^114^. The $\hbox{LaCoO}_3$ band gap given by LDA+*U* in our work^16^ was 1.94 eV, which is significantly larger than the experimental band gap of 0.5 eV^115,116^. However, these experiments reported a non-magnetic state, whereas we found the AFM state for a theoretical defect-free material. The reasons of disagreement between theory and experiments on the magnetic ground state of $\hbox{LaCoO}_3$ remains a subject of active research. Regarding the gap, we found that the band gap of the NM state of $\hbox{LaCoO}_3$ is 1.390 eV by LDA+*U*, which is closer to the experimental value.

Among the bulk structures, only $\hbox{LaMnO}_3$ has small density of states (DOS) around the Fermi energy, in agreement with an experiment^111^. They found that states exist around the Fermi level originating mainly from $e_{g}$ orbitals of the *d*-shell in Mn. They also observed that a large splitting exists between $t_{2g}$ and $\hbox{e}_g$ orbitals, and a small splitting is also in the $e_g$ orbitals. They explained that the small splitting is attributed to the Jahn–Teller distortions of the octahedral crystal field. Consequently, a significantly smaller band gap exists for $\hbox{LaMnO}_3$ than for the other two perovskites in our results. Our results show that point defects can turn $\hbox{LaMnO}_3$ metallic. Figure 4b and c indicate that antisite defects (oxygen vacancies) yield *p*-type (*n*-type) conductivity: antisite defects (oxygen vacancies) cause the Fermi energy to shift toward the valence (conduction) band. The shift of Fermi energy with charge neutral oxygen vacancies may be because of reduction of the system. Formation of +2 charged oxygen vacancies may inversely shift the Fermi energy toward the valence band. For $\hbox{LaFeO}_3$ and $\hbox{LaCoO}_3$, the antisite defects create defect energy levels in the band gap of the bulk structure. As a result, the band gap is reduced from 2.37 to 0.65 eV for $\hbox{LaFeO}_3$ and from 1.92 to 1.11 eV for $\hbox{LaCoO}_3$. The oxygen vacancies also narrow the band gap of $\hbox{LaCoO}_3$ from 1.92 to 1.12 eV and make $\hbox{LaFeO}_3$ conductive, producing an isolated DOS peak around the Fermi energy; the experimental disappearance or narrowing of the band gap could be evidence of formation of point defects.

## Conclusion

We studied the charge neutral antisite defects and oxygen vacancy formation energies of $\hbox{LaMO}_3$ (M = Mn, Fe, and Co) by FNDMC. Our calculations predicted a relative abundance of antisite defects for the cases of M = Mn and Fe, comparable with or higher than the oxygen vacancies, at the M-rich and O-poor conditions.

The transition metal atoms studied have significantly fewer electrons (25, 26, and 27) than La (57). Therefore, the presence of antisite defects should be difficult to observe in transmission electron microscopy experiments because the presence of antisite defects would be masked by the La atoms on the same column. However, we found that antisite defects affect the electronic and magnetic properties of the perovskite host. Our PDOS analyses showed that the antisite defects make the $\hbox{LaMnO}_3$ metallic introducing holes and energy levels inside the band gap of $\hbox{LaFeO}_3$ and $\hbox{LaCoO}_3$. Mid gap levels could be a signal that antisite defects have formed in experiments. Our FNDMC calculations also showed that the antisite defects have local magnetization.

## Supplementary Information

Supplementary Information.

## Data Availability

The calculation data for the results in this study is available from the corresponding authors on request.
