# Supplementary material for: Existence of La-site antisite defects in LaMO3 (M = Mn, Fe, and Co) predicted with many-body diffusion quantum Monte Carlo
Source: Sci Rep. 2023 Apr 25;13:6703. doi: 10.1038/s41598-023-33578-1 (PMC10130183; doi:10.1038/s41598-023-33578-1)
Supplement: Supplementary file 1 — Supplementary Information. [file 41598_2023_33578_MOESM1_ESM.pdf]

Supporting information: Existence of La-site antisite defects in $LaMO_3$ (M = Mn, Fe, and Co) predicted with many-body diffusion quantum Monte Carlo

Tom Ichibha[1,*], Kayahan Saritas[1], Jaron T. Krogel[1], Ye Luo[2], Paul R. C. Kent[3], and Fernando A. Reboredo[1,+]

[1]Materials Science and Technology Division, Oak Ridge National Laboratory, Oak Ridge, TN 37831, USA
[2]Computational Sciences Division, Argonne National Laboratory, Argonne, IL 60439, USA
[3]Computational Sciences and Engineering Division, Oak Ridge National Laboratory, Oak Ridge, TN 37831, USA
[*]A corresponding author; ichibha@icloud.com
[+]A corresponding author; reboredofa@ornl.gov

This manuscript has been authored by UT-Battelle, LLC, under contract DE-AC05-00OR22725 with the US Department of Energy (DOE). The US government retains and the publisher, by accepting the article for publication, acknowledges that the US government retains a nonexclusive, paid-up, irrevocable, worldwide license to publish or reproduce the published form of this manuscript, or allow others to do so, for US government purposes. DOE will provide public access to these results of federally sponsored research in accordance with the DOE Public Access Plan (http://energy.gov/downloads/doe-public-access-plan).

S1 Further details of FNDMC calculation conditions

Some minor differences exist among the calculation settings of (1) $LaMnO_3$ and $LaFeO_3$ and (2) $LaCoO_3$ because we compiled the results of two independent calculations in this paper. For the case (1), we used the hybrid orbital representation[11]. The plane waves were projected to two types of basis sets: (i) the spherical core region was expanded in products of angular momentum eigenfunctions and radial functions, $\sum Y_{l,m} v_{l,m}(\rho)$, whereas (ii) the interatomic region was replaced by B-splines. This approach facilitates the FNDMC calculation of large systems by significantly reducing memory requirements and can provide modest computational savings by slightly improving orbitals and reducing variance. We used twist-averaging boundary conditions conditions[12] to estimate the one-body finite size error. The twist grid size was equivalent to or larger than $2\times2\times2$ for ~80 atoms. We confirmed that the total energy of $LaMnO_3$ was converged within 2 meV/atom with this grid size with DFT. We used size extrapolation techniques[13] to estimate the two-body finite size error with ~40- and ~80-atom simulation cells. To determine the total magnetizations or optimal Hubbard U values for the perovskites, we used smaller total walker populations (≥476), but the population control error was confirmed to be 0.0203 eV/f.u. for $LaMnO_3$ and 0.0236 eV/f.u. for $LaFeO_3$. For the case (2), we used B-spline basis sets. The twist-averaging boundary conditions were used, and the grid size was $3\times3\times3$. We used multiple simulation cell sizes to perform the size extrapolation for the two-body finite size error. We confirmed that the extrapolated energies with and without the model periodic Coulomb (MPC) correction agreed with each other for every system, as shown in Figure S1.

Table S1. Experimental and FNDMC cohesive energies (eV/atom).

System	E_{coh}/expt. (eV/f.u.)	E_{coh}/FNDMC (eV/f.u.)
La_2O_3	7.03[1]	6.84(1)[1], 7.06(1)[2]
$LaFeO_3$	6.16[2,3]	6.24(2)
Fe	4.28[4]	4.00(5)
FeO	4.86[1]	4.82(3)
Fe_2O_3	5.03[5]	5.50(2)
$LaMnO_3$	6.06[6]	5.79(2)
MnO	4.75[7]	4.77(3)
MnO_2	4.51[7]	4.21(2)
O_2	2.56[8]	2.47(2)
CoO	4.72[9,10]	4.75(1)

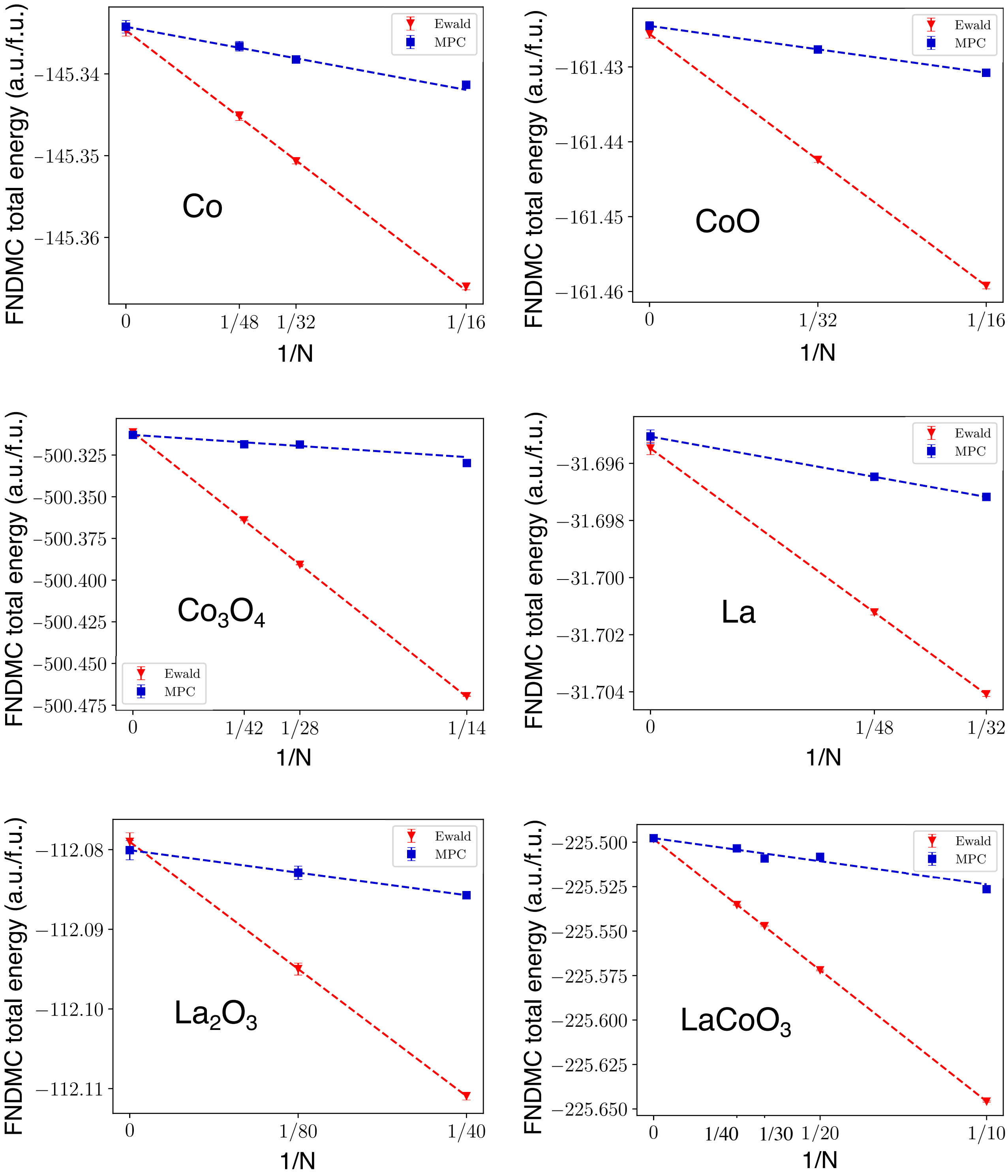

Figure S1. FNDMC size extrapolation for the systems used to calculate the chemical potentials for the $LaCoO_3$ defect formation energies.

S2 Coheisve energies

The experimental and FNDMC cohesive energies are listed in Table S1. The values correspond to the results in Figure 1 in the main text.

S3 Geometry effect for defect formation energies

Atomic structure optimizations are not yet practical with FNDMC because force calculations[14,15] are too expensive to be applied to isolated defect calculations. Therefore, DFT was used for the geometry optimization. To assess the defect formation energy's sensitivity to the given geometry, we compared the energies of two structures by using different functionals: one is relaxed with Perdew–Burke–Ernzerhof (PBE) and the other with strongly constrained and appropriately normed (SCAN), for $LaMnO_3$ with oxygen vacancies. The resulting energy differences were smaller than 4 meV. Therefore, we concluded that the method used to relax the atomic positions would not significantly influence the defects' formation energy.

(a) $LaFeO_3$ bulk (b) $LaFeO_3$ with antisite defect (c) $LaFeO_3$ with oxygen vacancy

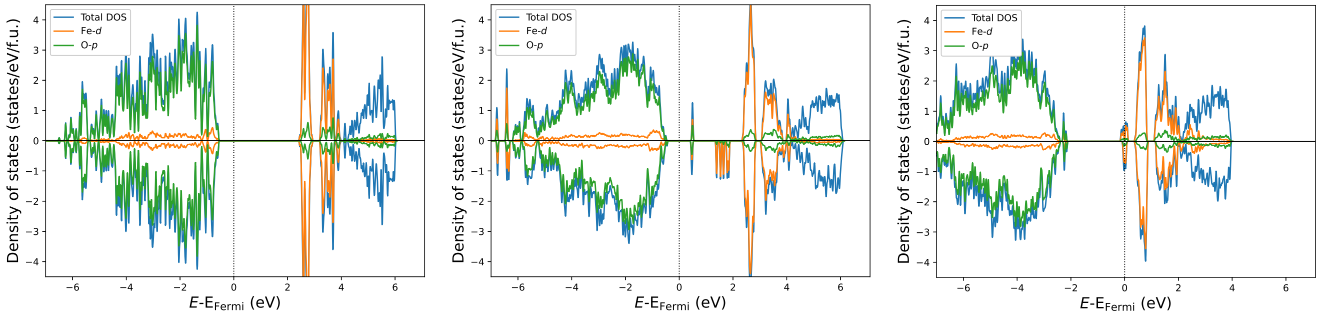

Figure S2. Densities of states of $LaFeO_3$ with (a) no defects (bulk), (b) antisite defects, and (c) oxygen vacancies.

(a) $LaCoO_3$ bulk (b) $LaCoO_3$ with antisite defect (c) $LaCoO_3$ with oxygen vacancy

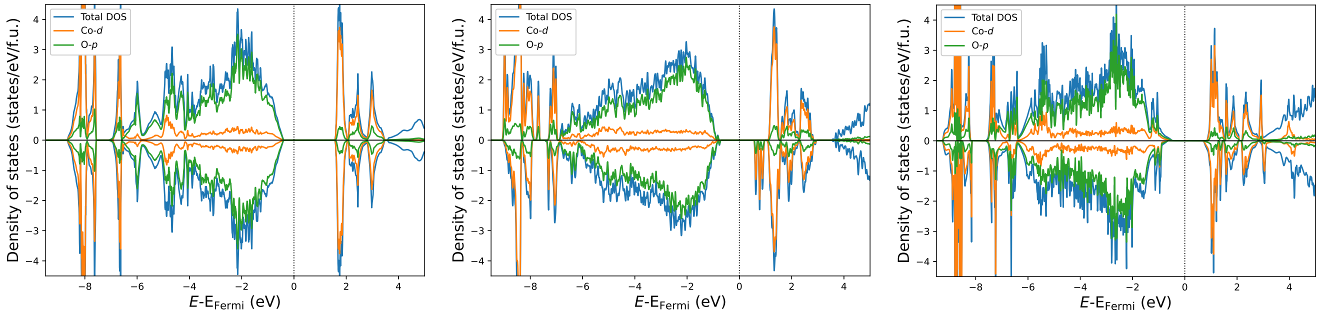

Figure S3. Densities of states of $LaCoO_3$ with (a) no defects (bulk), (b) antisite defects, and (c) oxygen vacancies.

S4 Density of states for $LaFeO_3$ and $LaCoO_3$

The density of states of $LaFeO_3$ and $LaCoO_3$ bulks and defected structures with antisite defects and oxygen vacancies are shown in Figures S2 and S3. Antisite defects narrow the band gap from 2.37 to 0.65 eV for $LaFeO_3$ and from 1.92 to 1.11 eV for $LaCoO_3$. Oxygen vacancies also narrow the band gap of $LaCoO_3$ from 1.92 to 1.12 eV and make $LaFeO_3$ conductive by creating defect states around the Fermi energy.

S5 Electron and hole doping effects for the defect formation energies

Electron (hole) doping increases (decreases) the Fermi energy. This could change the defect formation energies. For the $LaFeO_3$ case, antisite defects reduce and oxygen vacancies vanish the band gap, so electron doping could stabilize the defected structures compared to the bulk structure: the defect formation energies will be decreased. Hole doping could relatively stabilize the structure with oxygen vacancies because the Fermi energy could move from the defect levels to the valence band maximum.

For $LaCoO_3$, electron dopoing could reduce the defect formation energies with the same analogy as $LaFeO_3$. On the other hand, for $LaMnO_3$, electron and hole doping would not significantly affect the defect formation energies, because the band gap of the bulk structure is tiny and the defected structures are both metals.

References

1. Chase, M. W. J. NIST-JANAF thermochemical tables (American Institute of Physics, 1998).
2. Cheng, J., Navrotsky, A., Zhou, X.-D. & Anderson, H. U. Thermochemistry of $La_{1-x}Sr_xFeO_{3-\delta}$ Solid Solutions ($0.0 \le x \le 1.0$, $0.0 \le \delta \le 0.5$). Chem. Mater. **17**, 2197–2207, DOI: 10.1021/cm048613o (2005). https://doi.org/10.1021/cm048613o.
3. Cheng, J., Navrotsky, A., Zhou, X.-D. & Anderson, H. U. Enthalpies of Formation of $LaMO_3$ Perovskites (M = Cr, Fe, Co, and Ni). J. Mater. Res. **20**, 191–200, DOI: 10.1557/JMR.2005.0018 (2005).
4. Kittel, C. Introduction to Solid State Physics (Wiley, 2004), 8 edn.
5. Catti, M., Valerio, G. & Dovesi, R. Theoretical study of electronic, magnetic, and structural properties of α-Fe_2O_3 (hematite). Phys. Rev. B **51**, 7441–7450, DOI: 10.1103/PhysRevB.51.7441 (1995).
6. Rodríguez-Carvajal, J. et al. Neutron-diffraction study of the Jahn-Teller transition in stoichiometric. Phys. Rev. B - Condens. Matter Mater. Phys. **57**, R3189–R3192, DOI: 10.1103/PhysRevB.57.R3189 (1998).
7. Harrison, W. A. Tight-binding theory of manganese and iron oxides. Phys. Rev. B **77**, 245103, DOI: 10.1103/PhysRevB.77.245103 (2008).
8. of Japan, T. C. S. Handbook of Chemistry: Pure Chemistry, 5th ed. Maruzen Publ. Co.,Ltd. (2004).
9. Jog, K. N., Singh, R. K. & Sanyal, S. P. Phase transition and high-pressure behavior of divalent metal oxides. Phys. Rev. B **31**, 6047–6057, DOI: 10.1103/PhysRevB.31.6047 (1985).
10. Glasser, L. & Sheppard, D. A. Cohesive Energies and Enthalpies: Complexities, Confusions, and Corrections. Inorg. Chem. **55**, 7103–7110, DOI: 10.1021/acs.inorgchem.6b01056 (2016). PMID: 27362373, https://doi.org/10.1021/acs.inorgchem.6b01056.
11. Ganesh, P. et al. Binding and Diffusion of Lithium in Graphite: Quantum Monte Carlo Benchmarks and Validation of van der Waals Density Functional Methods. J. Chem. Theory Comput. **10**, 5318–5323, DOI: 10.1021/ct500617z (2014).
12. Lin, C., Zong, F. H. & Ceperley, D. M. Twist-averaged boundary conditions in continuum quantum Monte Carlo algorithms. Phys. Rev. E **64**, 016702, DOI: 10.1103/PhysRevE.64.016702 (2001).
13. Ceperley, D. M. & Alder, B. J. Ground state of solid hydrogen at high pressures. Phys. Rev. B **36**, 2092–2106, DOI: 10.1103/PhysRevB.36.2092 (1987).
14. Nakano, K., Raghav, A. & Sorella, S. Space-warp coordinate transformation for efficient ionic force calculations in quantum Monte Carlo (2021). arXiv:2110.12234.
15. Tiihonen, J., Clay, R. C. & Krogel, J. T. Toward quantum Monte Carlo forces on heavier ions: Scaling properties. The J. Chem. Phys. **154**, 204111, DOI: 10.1063/5.0052266 (2021). https://doi.org/10.1063/5.0052266.
